# Population authentication of the traditional medicinal plant *Cassia tora* L. based on ISSR markers and FTIR analysis

**DOI:** 10.1038/s41598-018-29114-1

**Published:** 2018-07-16

**Authors:** Vikas Kumar, Bijoy Krishna Roy

**Affiliations:** Centre of Advanced Study in Botany, Institute of Science, Banaras Hindu University, Varanasi, 221005 India

## Abstract

*Cassia tora* is a plant of medicinal importance. Medicinal plants from different localities are believed to differ in their therapeutic potency. In this study, six populations of *C. tora* with different eco-geographical origins were investigated genotypically (ISSR) and phytochemically (FTIR) to establish an integrated approach for population discrimination and authentication of the origin of this medicinal herb. *CHS* gene expression analysis and determination of flavonoid content were carried out to substantiate the study. A total of 19 population-specific authentication bands were observed in 11 ISSR fingerprints. Authentication codes were generated using six highly polymorphic bands, including three authentication bands. FTIR spectra revealed that the peaks at wavenumber 1623 cm^−1^ (carbonyl group) and 1034 cm^−1^ (>CO- group) were powerful in separating the populations. These peaks are assigned to flavonoids and carbohydrates, respectively, were more intense for Ranchi (highland) population. Variation in the transcript level of *CHS* gene was observed. The findings of FTIR and RT-PCR analyses were in agreement with the TFC analysis, where, the lowest amount of flavonoids observed for Lucknow (lowland) population. All the populations of *C. tora* have been authenticated accurately by ISSR analyses and FTIR fingerprinting, and the Ranchi site was observed to be more suitable for the potential harvesting of therapeutic bioactive compounds.

## Introduction

The therapeutic potential of plants has been utilized in traditional medicines such as Chinese, Ayurveda, Siddha, and Unani etc. Being relatively nontoxic and easily affordable, there has been resurgence in the demand for medicinal plants^1^. *Cassia tora* L. *Syn*. *Senna tora* (L.) Roxb. verna. Chakwad, commonly known as sickle senna, belongs to family Caesalpiniaceae (Subfamily: Caesalpinioideae, tribe: Cassieae, sub tribe: Cassiinae). It is the wild annual herbal crop, indigenous to palaeotropical region (Africa and Asia to eastward Polynesia) and distributed throughout the tropical and sub-tropical regions of the world^2–4^. The plant is widely consumed as a potent source of sennosides (laxative), and enlisted in the World Health Organisation’s ‘*List of Essential Medicines*’^5^. It's medicinal potentials have been described in the traditional Chinese medicine (TCM) and Ayurvedic practices with the special reference to cure psoriasis and other skin degenerative disorders^6^. In addition to this, the plant was also used traditionally to cure diabetes, dermatitis, constipation, cough, cold and fever, etc^7^. The plant harbors anti-proliferative, hypolipidemic, immunostimulatory, and anticancerous properties^8^. Earlier, It has been reported that *C. tora* possessed a large amount of flavonoids, the potent antioxidants^9,10^. The production of flavonoids in plants is linked with the expression of chalcone synthase (*CHS*) gene encoding *CHS* enzyme which is the first committed enzyme in flavonoid biosynthesis^11,12^. *CHS* is ubiquitous to higher plants and belongs to the family of polyketide synthase (PKS) enzymes (known as type III PKS). It is believed to act as the central hub for the enzymes involved in the flavonoid pathway^12,13^. The expression of *CHS* gene is the important step in the biosynthesis of flavonoids^14,15^ and *CHS* transcription is regulated by endogenous programs in response to environmental signals^16^. Plant samples from different geographical origins have different biochemical compositions due to variations in the environmental conditions and genetic reasons^17–19^. Therefore, it is crucial to identify the medicinal herb at the locality level.

The general approach to identification is dependent on morphological, anatomical, and chemical features, but such characteristics are often affected by environmental and other developmental factors during plant growth^20^. Additionally, medicinal plants are processed for use as crude drugs, which affect many morphological and anatomical characteristics, as well as resulting in changes in some chemical constituents^21^. Therefore, it is difficult to identify the crude herbs through anatomical and chemotaxonomical studies. The established DNA barcoding approaches to authenticate plant species were based on either a short, and standardized DNA sequence region or DNA polymorphism using genetic markers like ISSR, and SNP etc.^22–24^. In plants, variations among the plastid gene sequences, for example, *rbcL, matK* and *trnL* genes, and ITS regions were used in identification, population discrimination and authentication of various plant species^25–29^. However, the lack in the prior information of genomic regions and low evolutionary rate of change in the coding regions are serious limitations to such analysis. A DNA based polymorphism assay may be the suitable alternative for the population authentication of herbal medicines. Earlier studies based on genetic markers (RAPD, SCAR and ISSR etc.) could significantly identify the plant populations^30,31^, however ISSRs were found highly reproducible, more variable and efficient than the currently used DNA marker^32–34^ in being the more robust to even slight changes in DNA concentrations. In addition, they retained the benefits over other PCR-based techniques such as the need for very little template material. Nevertheless, DNA genotyping also has limitations such as within species variation. Furthermore, the technique does not reveal the composition of the active ingredients or chemical constituents. DNA remains the same irrespective of the plant part used, while the phytochemical compositions may vary with the plant parts used, physiology, and the environment^19,21^. Therefore, proper integration of DNA based techniques like ISSRs and analytical tools like FTIR for chemo-profiling would be more efficient to authenticate the population and will lead to the development of a comprehensive system of characterization that can be conveniently applied at the industry level for quality control of herbal drugs.

Therefore, the aim of this study was focused upon a) development of molecular markers to distinguish the test populations of *C. tora* and b) discriminate the same populations based on the variability in phytochemical (mainly flavonoid) content. For the aforementioned purposes, we used the ISSRs and FTIR as the rapid and efficient techniques. In addition, we also employed a qPCR based approach to test the expression level of *CHS* gene and total flavonoid content (TFC) analysis to substantiate the study. To the best of our knowledge, this study is the first attempt of its type.

## Results

### Amplified products

**Figure 1:**
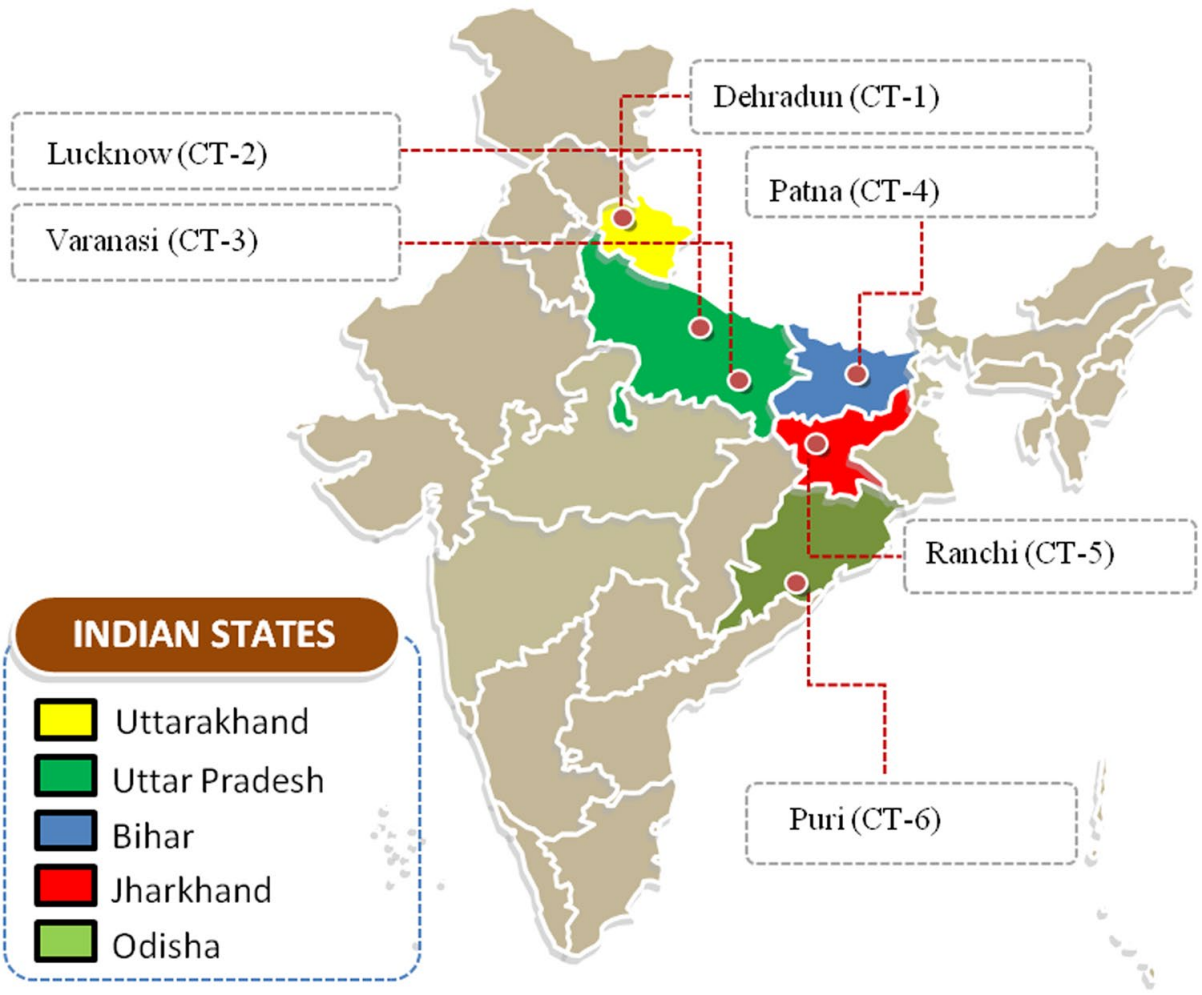
Collection sites of *Cassia tora*. This image is prepared with the help of an editable map of India (Source: https://yourfreetemplates.com).

**Figure 2:**
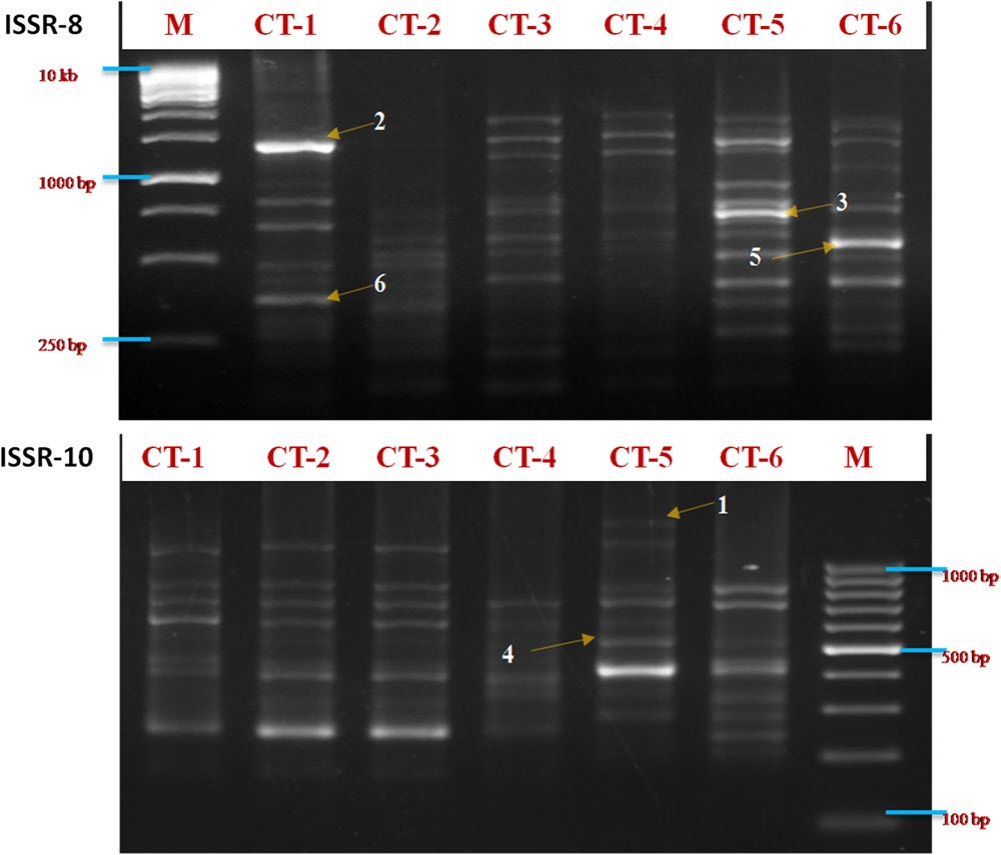
Agarose gel images showing amplification pattern of six *C. tora* populations obtained by ISSR-8 and ISSR-10 indicating selected polymorphic bands for the authentication of *C. tora* population.

A total of 130 clear and reproducible bands were amplified from six populations of *C. tora* which were collected from different geographical locations (Fig. 1, Supplementary Table S1) using the 11 selected ISSR primers, of which, 118 were polymorphic (90.76%). The total number of loci varied from 31 to 54 per primer for all the populations (Table 1), with fragment size ranging from 200–3000 bp (Fig. 2). Among the samples, the CT-5 (Ranchi) population had the highest ISSR polymorphism (85.90%), while the CT-1 (Dehradun) population, the lowest (81.97%). The lowest genetic distance, based on Jaccard's coefficient, was between CT-3 (Varanasi) and CT-4 (Patna) populations, and the highest between CT-2 (Lucknow) and CT-6 (Puri) (Supplementary Table S2). ISSR fingerprinting of six populations using primer ISSR-8 and ISSR-10 is shown in Fig. 2.

**Table 1:** Details of markers selected for the study and their amplified products.

S. No.	ISSR Primers	Sequence (5′-3′)	Annealing temperature (°C)	Size range of Amplicons	No. of amplified bands	Polymorphic loci	Polymorphism (%)
1.	ISSR5	(GTG)_5_	52	200 bp–2.8 kb	13	12	92.30
2.	ISSR7	(AC)_8_G′	48	100 bp–2.9 kb	17	17	100
3.	ISSR8	(GA)_8_CT	52	150 bp–2.8 kb	15	15	100
4.	ISSR10	(CA)_7_CC	48	200 bp–2.5 kb	11	9	81.82
5.	ISSR11	(CA)_7_CG	48	100 bp–2 kb	10	9	90
6.	ISSR13	(GT)_7_CG	48	250 bp–2.8 kb	9	8	88.89
7.	ISSR16	(GTG)_4_GAC	52	200 bp–2.1 kb	9	9	100
8.	ISSR17	(AG)_8_G	49.5	350 bp–2.1 kb	11	8	72.73
9.	ISSR21	(TC)_8_C	49.5	250 bp–2 kb	11	11	100
10.	ISSR22	(TC)_8_G	49.5	300 bp–1.1 kb	13	10	76.92
11.	ISSR25	(GA)_8_GT	49.5	250 bp–3 kb	11	10	90.91
			Total		130	118	90.76

### Development of specific authentication markers for *Cassia tora* population

From the DNA fingerprints, based on ISSR primers, highly polymorphic bands were selected for the population identification. Total nineteen (14.62%) specific authentication bands observed which were present in one population but absent in other (Table 2). Total six highly polymorphic bands were selected as authentication bands to identify the *C. tora* population (Fig. 2). These were scored as zero (0) and one (1), based on the absence and presence of the polymorphic bands in the rest of the population (Table 3).

**Table 2:** Specific authentication bands (kb) from six *C. tora* populations.

S. No.	Primers	*Cassia tora* population					
		CT-1	CT-2	CT-3	CT-4	CT-5	CT-6
1.	ISSR5	1					
2.	ISSR7			0.7	0.52		
3.	ISSR8					0.7	0.6
4.	ISSR10					2.5	
5.	ISSR11	0.9				0.7	
6.	ISSR13	0.3				2.8	
7.	ISSR16	0.45				0.8	
8.	ISSR17						0.75
9.	ISSR21			0.3		0.9	
10.	ISSR22					0.55	
11.	ISSR25	0.25	0.35		3

**Table 3:** ISSR genotypes of six *C. tora* populations.

Population code	ISSR authentication markers						Authentication code
	1	2	3	4	5	6	
CT-1	0	1	1	0	1	1	011011
CT-2	1	0	1	0	0	0	101000
CT-3	1	1	0	1	1	1	110111
CT-4	1	1	1	1	1	0	111110
CT-5	1	0	1	1	0	1	101101
CT-6	0	1	1	0	1	0	011010

### FTIR analysis

**Figure 3:**
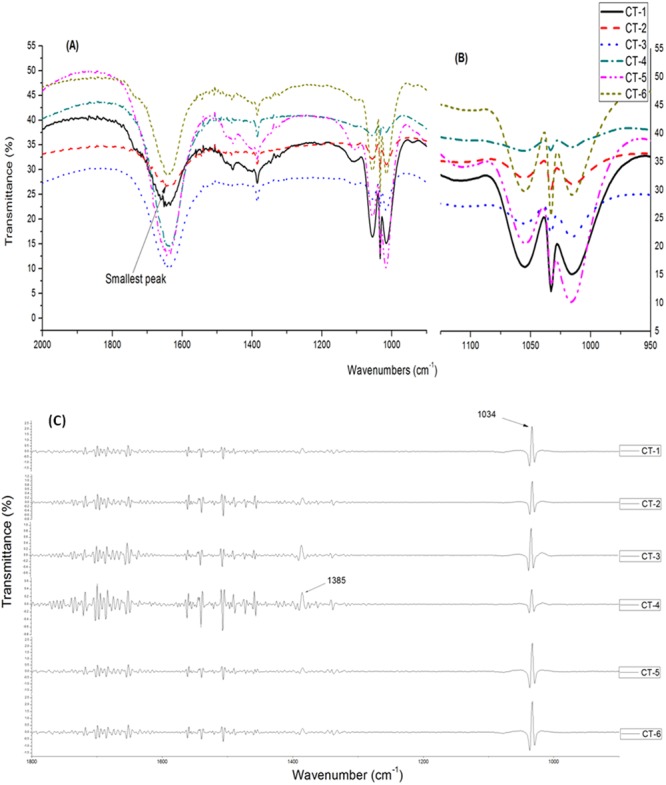
FTIR-spectra of six populations of *C. tora* collected from Dehradun (CT-1), Lucknow (CT-2), Varanasi (CT-3), Patna (CT-4), Ranchi (CT-5), and Puri (CT-6). (**A**) A portion of phytochemically important region (1800-900 cm^−1^); (**B**) An enhanced view; (**C**) Secondary derivatives of FTIR-spectra of six population of *C. tora*.

FTIR spectra of six *C. tora* populations having different geographic origins (Supplementary Table 1) are depicted in Fig. 3A. Though, the repeat measurements from one region showed no significant difference in the spectra hence, only one profile was given for a population (Fig. 3A). The spectra showed broadly similar transmittance patterns for all the tested populations. Several prominent peaks in spectra indicated the presence of specific functional groups in common among all the populations. The result showed high absorbance at wavenumber region of 3400–3200, 3200–2800, 1800–1500 and 1100–950 cm^−1^. The fingerprint region, 2000–900 cm^−1^ was chosen for further analysis. Besides the similar transmittance pattern observed in spectra for all the populations, the distinct intensity of prominent peaks were observed between different populations i.e. CT-1 (Deharadun) and CT-5 (Ranchi) populations which showed intense peaks compared to rest of the populations (Fig. 3A,B). According to geographic elevation, we divided the all populations into two groups, highland (<500 m) and lowland (>500 m), and performed student’s t-test to determine the level of significance of difference between their transmittance patterns, and it was highly significant (P>0.01). However, smaller differences in-between populations were most difficult to resolve as many spectra overlapped. Thus secondary derivatives (SD)-IR were used to enhance the resolution and to amplify small differences in the IR spectra. The SD-IR spectra were more idiosyncratic among the different populations. The spectra shown in (Fig. 3C), revealed the three prominent peaks assigned to wavenumbers 1384 and 1034 cm^−1^. Peaks at 1034 cm^−1^ were more intense, and is assigned to carbonyl group (carbohydrate region)^35^ (Supplementary Table S3). However, the fluctuation between peaks intensities could be noticed easily throughout whole spectra.

### Multivariate analysis

**Figure 4:**
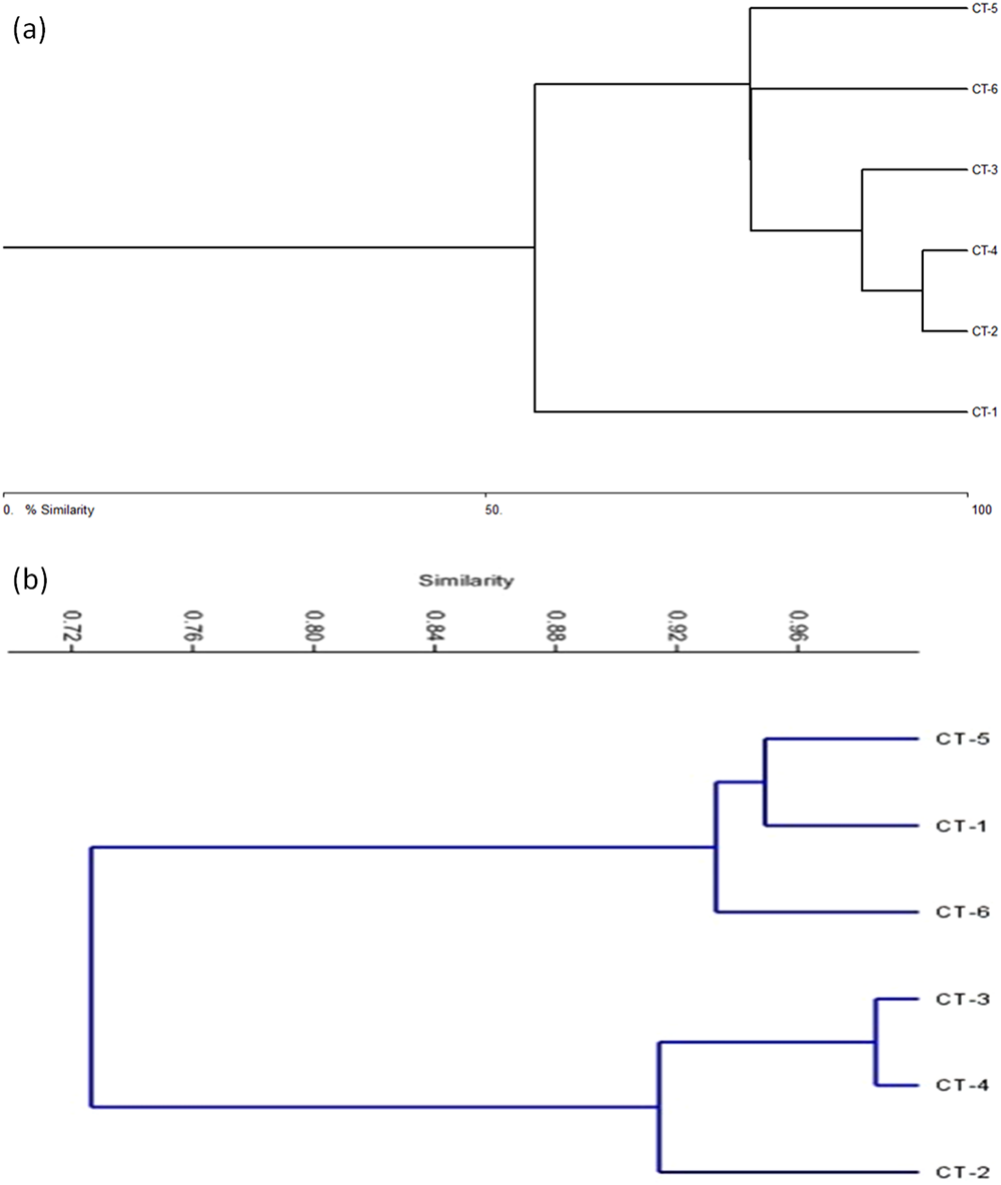
ISSR data (**a**) and FTIR absorbance (**b**) based clustering of *C. tora* populations.

**Figure 5:**
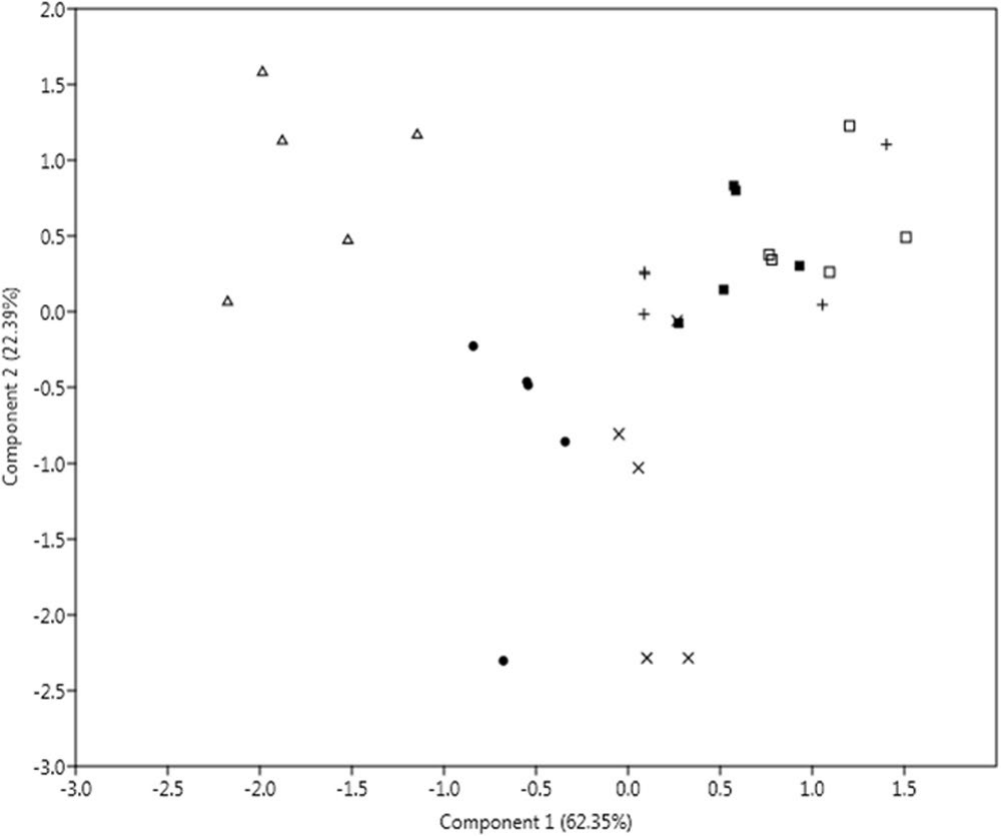
PCA model based on transmission data analysed by IR spectra of the six accessions of *C. tora*. The percentage of variation of the data explained by each component is provided in the plot. Dot, plus, square, triangle, X and fill square symbols indicated CT-1, CT-2, CT-3, CT-4, CT-5 and CT-6 populations, respectively.

Cluster analysis was performed using polymorphic data generated by ISSR analyses to observe similarity among the *C. tora* populations (Fig. 4a). The visual observation of FTIR spectra showed no significant difference in the characteristic transmittance bands among tested populations, and the intensity of peaks at certain wavenumbers did not differ among each other especially at fingerprint region 2000–900 cm^−1^. Therefore, it is more practical to incorporate statistical method for the aid of interpreting the measurements obtained. Since the authentication of different geographical origins of the herb based on the slight differences among particular absorption bands is too subjective, the results may vary among the analysts as reported earlier. The principal component analysis (PCA) and Pearson’s correlation were carried out between the selected spectral regions (2000–900 cm^−1^) (Fig. 4b). PCA revealed 62.35% variance for principal component (PC)-1 and 22.39% for the PC-2 (Fig. 5).

### Total flavonoids content

**Figure 6:**
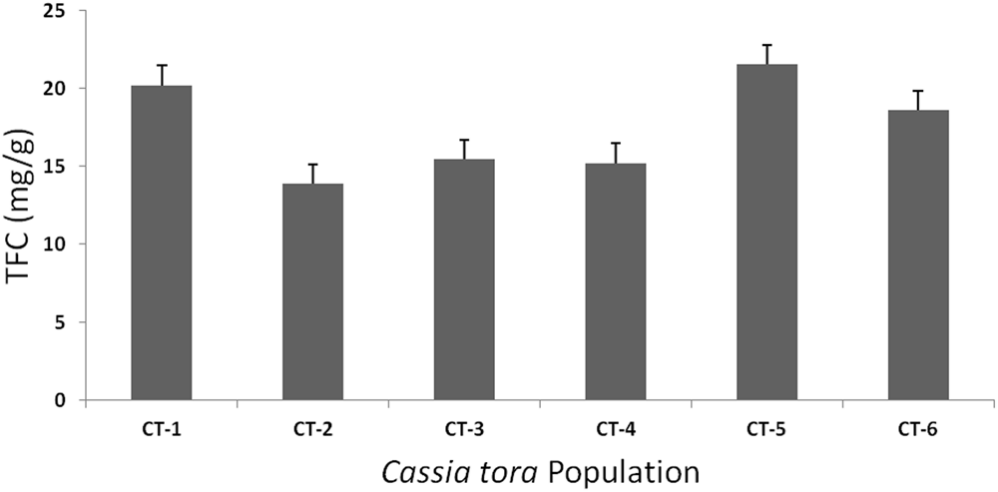
Total flavonoid content (TFC) of six *C. tora* populations.

The total flavonoids content (TFC) in extracts, was determined using the formula (y = 0.005x + 0.085), derived from the calibration curve, and expressed as mg/g leaf dry wt (in terms of quercetin equivalent). High yield (21.53 mg/g) of TFC was observed for CT-5 (Ranchi) population. However, lowest yield (13.87 mg/g) was found in CT-2 (Lucknow) population (Supplementary Table S4, Fig. 6).

### CHS gene analysis

**Figure 7:**
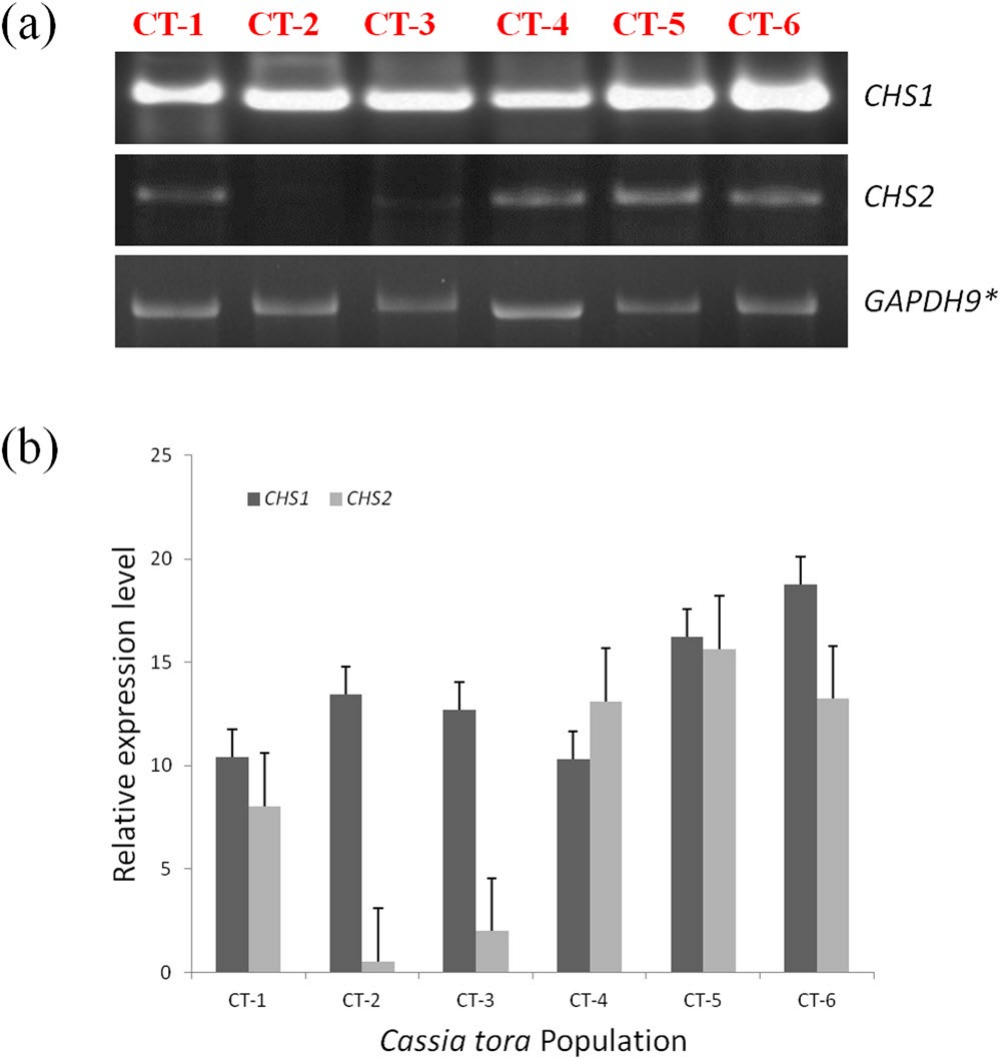
RT-PCR of Chalcone synthase (CHS) gene: (**a**) visualization of amplified genes (**b**) Relative transcript expression level in *C. tora* leaves of the six accessions was determined. Error bars indicate the standard error of the mean ± SE of three replicate measurements. (*control gene).

*CHS1* and *CHS2* genes of *C. tora* from six different geographic origins were analysed by semi quantitative RT-PCR. *CHS1* gene showed clear variation in the relative expressions among the populations. The lowest transcript level was observed in the CT-4 (Patna) population and the high quantity of transcripts was observed for the CT-6 (Puri) population. *CHS2* analysis disclosed the lowest transcript level for CT-2 (Lucknow) population however, relatively high expression was observed in CT-5 (Ranchi) population (Fig. 7). The result indicated the occurrence of variable transcript level of the *CHS* gene among *C. tora* populations of different geographical regions.

## Discussion

Traditionally, *C. tora* is claimed to be useful in the treatment of psoriasis and other skin diseases^6,7^. Earlier, antipsoriatic activity of three flavonoids, namely quercetin-3-O-Dglucuronide, luteolin-7-O-glucopyranoside and formononetin-7-O-D-glucoside from *C. tora* leaves were investigated using UV-B induced photodermatitis model, revealed the significant (p < 0.01) percentage reduction of relative epidermal thickness compared to positive control^6,36^. The environment has influences on plant development and metabolism, and can alter the plant’s chemical compositions and therapeutic potency^18,19^. Therefore, selection of the genuine populations for potent application has become the key issue in the modernization of traditional medicines. It is, however, difficult to authenticate genuine from among the wild populations accurately using the conventional techniques, as they are similar in morphological, anatomical characteristics, and also, sometimes in chemical components too^37^. Hence, the precise identification and authentication of genuine population is a prerequisite for the chemical and pharmacological investigations of traditional medicines, as well as for their clinical applications.

In this study, we have investigated the qualities of 25 ISSR primers to generate polymorphic DNA fragments among which eleven primers were selected. Total 130 bands were obtained in fingerprints by 11 ISSR markers, among which, 118 bands were polymorphic (90.63%) which indicates that the simple sequences were abundant and highly dispersed throughout the genome of *C. tora*, and highly polymorphic. The results are consistent with the view point that level of genetic diversity as affected by the species distribution^38^. At the same time, we have detected 19 population-specific authentication bands, and established ISSR authentication codes involving three authentication bands for the each population of *C. tora* (Tables 2 and 3), which efficiently enhanced population authentication and validated the ISSR-PCR technique as the efficient marker system to be utilized to construct DNA fingerprints and to authenticate the plant populations. Earlier, ISSR authentication codes had been generated to authenticate the various medicinal plant populations like *Dendrobium officinale*^30^ and rhubarb^39^. The high polymorphism among populations also points out the rich genetic variability of *C. tora*. The lowest polymorphic bands (50) for Dehradun population proved the declination of genetic functions of a species at higher altitude and low temperatures^40,41^. Bary-Curtis cluster analysis of all populations favored the above findings and split Dehradun population from the rest (Fig. 4a). However, Ranchi population had highest polymorphism (85.9%) indicating that genetic exchange and differentiation of populations increased slightly at the higher elevation, probably due to extensive gene flow at the altitudes^42^.

FTIR has been proven to be an accurate, fast and simple method for phytochemical screening^43^. It provides more information through the fingerprint regions of herbal medicines, rendering the technique direct and simple^21,44^. Previously, FTIR has been used in identification and population discrimination studie^35,45,46^. Samples from the different populations can be discriminated based on the functional group absorption. Peak absorbance at the particular wavenumber is presented in (Supplementary Table S3). The IR spectra of wave-region (3400–3200, 3200–2800, 1800–1500 and 1100–950 cm^−1^) were similar for all the populations with the variable intensity, which implies the presence of similar major chemical components in all the samples obtained from different locations. Previously, the methanolic extract of *C. tora* had been analyzed using FTIR, and flavonoids were reported as a major phenolic component in the plant^10^. Carbonyl (>C=O) group constitutes the functional group of flavonoid, in which, stretching vibrations in carbonyl compounds lie between 1750-1600 cm^−1^ of mid-IR^47^. The sharp peaks in this region indicated that the all extracts were flavonoid-rich. Nevertheless, the high absorbance of O-H, C=C, and C-O-C functional group in methanol extracts of the leaf indicated that the phenol and flavonoids could be the dominant compounds^10^. The absorbance by C-O, C-H, C=C, C=O, and C-N functional groups between 1800–900 cm^−1^ are indicative of benzene, aldehyde and carbohydrate groups (Supplementary Table S3). Stronger absorption peaks in these regions (Fig. 3) for CT-1 (Dehradun) and CT-5 (Ranchi) sample suggests a high amount of such compounds among highland populations^21^. Chan *et al*. (2007)^48^ had also reported the high amount of phenolics in highlands population of ginger. The high flavonoid content in Dehradun and Ranchi populations may be due to ecological stresses like a decrease in soil moisture and nutrients availability or the decreasing temperature^49^. These stresses could have led to oxidative damages, and as the antagonistic response, plants synthesized abundant antioxidants especially the phenolics^50^.

The analysis based on SD-IR provides a better way to distinguish the populations when the peaks overlapped as SD-IR spectra enhanced the apparent resolution and amplified the tiny differences in the IR spectrum^45^. In this study, several peaks overlapped together at a single wavenumber making their appearance incoherent; therefore, SD-IR was used to resolve the peaks and to reveal the weaker spectral features (1800-800 cm^−1^) for interpreting the components with a low concentration and weak absorption peaks^44^. Results of SD-IR of the *C. tora* leaf illustrated the two distinct and sharp peaks in-between the wave region (1500-900 cm^−1^) assigned to benzene (1384 cm^−1^) and carbonyl group (1034 cm^−1^) (Fig. 3C), which clearly exhibited the presence of phenolics and carbohydrates in all the six populations with variations in their constituents. Peaks between wavenumbers (1500- 1200 cm^−1^) and (1100–900 cm^−1^) assigned to the carbohydrate region^21,35^. Patna population had the highest absorption peak at 1384 cm^−1^, and lowest at 1034 cm^−1^. Low-intensity peak indicated the presence of low amount of the carbohydrates of this population compared to all the populations. According to the above findings, Patna population could be easily discriminated from rest of the populations. Earlier, similar studies had also been used for population discrimination of *Polygonum minus*, species discrimination in between *Tephrosia tinctoria* and *Atylosia albicans*, and identification of genuine American ginseng population^21,45,46^.

Cluster analysis based on similarity matrix successfully discriminated all the populations into two separate groups, one with the populations CT-1, CT-5, and CT-6; and another with CT-2, CT-3 and CT-4 (Fig. 4b). Both the groups were highly (28%) dissimilar. Dehradun and Ranchi populations were 94% similar while Varanasi and Patna populations 98% similar, indicating that they shared almost the common phytochemical constituents. However, Puri and Lucknow populations distantly placed in their cluster showed the difference in chemical compositions and could be easily discriminated. PCA analysis also showed a disparity among the populations (Fig. 5) and separated them with the varying eco-geographical features especially in-between Highland (Dehradun and Ranchi) and Lowland populations. Such variations in absorbance were linked to quality and quantity of the phytochemical constituents may also be due to the altitude effect^51^. Higher altitude like that of Dehradun, exposed the plants to intense solar radiation than the rest (Supplementary Table S1) and can be inversely affected by temperature, and therefore, plant defense system produces excessive phenolics to protect against photo-damage^52^.

In order to substantiate our study, we have also conducted the quantitative analysis of TFC. Among all the six populations, the highland populations (Ranchi and Dehradun) had highest flavonoid content (21.53 mg/g and 20.20 mg/g, respectively) followed by lowland populations (Fig. 6, Supplementary Table S4). The similar findings were also reported for the highlands population of ginger^48^. ISSR analysis also corroborated these findings where maximum polymorphism was observed for Ranchi population (85.9%). However, with relatively lower polymorphic DNA (81.9%) compared to other populations, Dehradun population estimated high amounts of flavonoids after Ranchi, and it might be due to the geographical elevation and other physical and physiological stresses^49,50^. Therefore, it is emphasized that Ranchi population produced secondary metabolites in greater abundance and is more genetically affluent than the others. Hence, this population can be better exploited for the germplasm conservation and breeding purposes. The high TFC content of Ranchi populations along with Dehradun and Puri populations (Fig. 6) could be correlated with the FTIR spectra (Fig. 3) and cluster analysis (Fig. 4b) where, these populations were clustered separately. Varanasi and Patna populations had comparatively larger and intense peaks at wave region 1750–1100 cm^−1^ than Lucknow populations, indicating these populations to be rich in flavonoids over the Lucknow population and that were also substantiated by TFC analysis (Figs 3 and 6). Dehradun populations showed the lowest DNA polymorphism, and clustered separately from rest of the population in accordance with indices of Bary-Curtis similarity, although contained good amounts of the flavonoids as per the analysis of IR and TFC data (Figs 3, 4a and 6). Based on the above results, this can be suggested that plants growing at relatively low temperatures (Supplementary Table1) might have high phenylalanine ammonia lyase activity, the key enzyme of phenylpropanoid pathway that possibly leads to the accumulation of flavonoids^53^.

Up regulated transcription of genes encoding enzymes involved in phytochemical biosyntheses, such as *CHS*, leads to increased phytochemical (i.e. flavonoids) concentrations in plants^11,54^. Genes encoding *CHS* constitute a multigene family in which the copy number varies among the plant species, and functional divergence appears to have repeatedly occurred^55^. *CHS* gene expression has been studied extensively in relation to flavonoids production in many plant species^14,15^. However, there are few reports about the CHS gene analysis in sub-tribe Cassiinae. Panigrahi *et al*. (2013)^56^ correlate the flavonoid content with the presence of *CHS* gene in between *C. laevigata* and *C. fistula*, and Samappito *et al*. (2013)^57^ studied the expression of *CHS* gene in *C. alata* roots and correlates their role in the synthesis of flavonoids. In our study, a preliminary attempt was made to access the expression level of *CHS* among all the tested populations of *C. tora* because this plant is rich in flavonoids which are a major source of bioactive compounds^10^. *CHS1* analysis showed higher transcript level for Ranchi and Puri populations compare to the rest (Fig. 7b). Earlier, it has been reported that CHS is constitutively expressed in plants but can also be subject to induced expression through light and temperature^58^. Therefore, higher expression of *CHS1* gene in Puri population might be due to the high geographical temperature (Supplementary Table S1), responsible for the larger production of flavonoids as observed in FTIR spectra and TFC analysis (Figs 3A and 6). *CHS2* showed lowest expression for Lucknow population (Fig. 3B), might be linked to the lesser production of flavonoids as observed in IR-spectra (Fig. 3A) with the smallest (low intense) peak in the flavonoid zone (1750-950 cm^−1^) and was also in agreement with TFC analysis (Fig. 6). Nevertheless, the higher transcript level of *CHS2* in Ranchi population (Fig. 3) was also in agreement with FTIR analysis and TFC. Thus it may be inferred that the variable transcript level of *CHS* gene might be responsible for the lopsided distribution of flavonoids^56,57^, among *C. tora* populations and proficient to discriminate the populations from different localities.

## Conclusions

ISSR fingerprinting was the suitable method for estimating the genetic differences among the populations and the authentication codes developed during analysis, will be helpful in differentiating the *C. tora* populations. However, FTIR spectrum analysis seemed appropriate to monitor the phytochemical variations among different *C. tora* populations. Both the techniques, ISSRs and FTIR established a very rapid, efficient and cost-effective technique to characterize the *C. tora* populations having different eco-geographical origins. *C. tora* population of Ranchi locality was genetically effluent comparatively rich in bioactive compounds, and hence this site would be most suited for the collection of germplasm and high amount potent bioactive compounds. Furthermore, we can also conclude that highland populations of *C. tora* produced certain secondary metabolites (flavonoids) in greater quantity than lowlands ones.

## Materials and Methods

### Plant material

*C. tora* plants were collected from their natural habitats in August 2014 at six different locations in India (Fig. 1). All the samples were identified using the morphological characters encrypted in the monograph and other relevant literature^9^. In addition to this, the plants were also authenticated by Prof. N. K. Dubey, taxonomist of the department of Botany, Banaras Hindu University (BHU), Varanasi, India. For the each population, herbarium specimen was prepared and deposited at the ‘Herbarium’ of the above-mentioned institution with the voucher specimen number (*Caesal/2014/1*). These taxonomically authenticated samples are referred to as Biological Reference Material (BRM)^59^. The plant collection sites with eco-geographical details are given in (Supplementary Table S1). Three individuals per population were taken with technical replicates for all the experimental analyses.

### DNA extraction

The genomic DNA was extracted from lyophilized young leaves using the cetyl trimethyl ammonium bromide (C-TAB) method of Wang (2010)^60^ with the given modifications. 30 mg of Polyvinylpyrrolidone (PVP) was added to remove polyphenols and extracted sample was treated with RNase (30 µg, 37 °C) for 30 min. DNA concentration and purity were determined by spectrophotometry (ND-2000, NanoDrop, USA) and electrophoresed on 0.8% agarose gels. The final concentration of each DNA sample was diluted to approx 20 ng/ml with Mili-Q water and stored at 20 °C till further use.

### PCR amplification

#### ISSR-PCR

PCR amplification was carried out in a total volume of 25 µl, containing 20 ng of template DNA, 2.5 µl 10X PCR buffer, 2.5 µl 25 mM MgCl_2,_ 2 µl 10 mM dNTPs, 0.32 mM primer, 2.5 unit of Taq polymerase, and Mili-Q water. The reactions were performed in a Mastercycler thermocycler (BioRad, USA). The program consisted of an initial denaturation at 94 °C for 4 min, followed by 35 cycles of 45 s at 94 °C, 45 s at 48–52 °C (depending on the primer), 2 min at 72 °C, and the final extension of 10 min at 72 °C. A negative control, with the template DNA omitted, was included in each PCR. Amplification products were electrophoretically separated at a constant voltage of 60 V for 3 h, in 1.5% agarose gels with 0.5X TAE buffer, stained with ethidium bromide and visualized under UV. The 100 bp and 1 kb DNA ladders were used to estimate the molecular size of the fragments. Twenty-five primers were tested to identify those that produced sharp and reproducible bands. Three individuals from each population of *C. tora* were randomly chosen for the experiment. The eleven primers selected for this study were used to amplify all the *C. tora* DNA (Table 1).

### CHS gene analysis

Total RNA was isolated from the leaf samples (100 mg) using TRIZOL reagent (GIBCO-BRL) as per instructions given in the manufacturers’ protocol. The total RNA was digested with DNase at 37 °C for 15 min and then reverse transcribed into cDNA using M-MLV Reverse Transcriptase RNaseH (Bio-Rad CFX-96^TM^ system). Primers were designed using the software Primer 3 (*CHS1* forward: 5′-AGCCAGTGAAGCAGGTAGCC-3′, *CHS1* reverse: 5′-GTGATCCGGAAGTAGTAAT-3′ and *CHS2* forward: 5′-AGCCAGTGAAGCAGGTAGCC-3′, *CHS2* reverse: 5′- GTGATCCGGAAGTAGTAAT -3′), referring to accessed sequences in the Genbank (Supplementary Table S5). The semi quantitative RT-PCR of selected genes was done according to Goto-Yamamoto *et al*. (2002)^61^. Triplet of all sample reactions were carried out and negative control of master mix in addition to primers was performed in all RT-PCR runs. *GAPDH* was taken as control because of its constitutive expression. The accuracy of primers was tested using genomic DNA of the plant as positive control. The intensities of the PCR products on agarose gels were quantified with the Gel Doc 2000 system and volume tool of the Quantity one software (BioRad, USA).

### Data analysis

The amplified products were scored in terms of the binary code as present (1) or absent (0), each treated as the unit character regardless of its intensity. Polymorphism at the population level was calculated as the ratio of polymorphic loci to the total number of loci scored in all accessions of the same population. The pair wise genetic distance matrix was computed using UPGMA, NTSYSpc version 2.02e (Supplementary Table S2).

### Sample preparation and extraction

Leaves of *C. tora* were selected for the preparation of extracts because they are more frequently used for medicinal purposes^62^. Leaves are generally more sensitive to changes in the environmental factors than other organs, and the difference in their traits has been used to classify plants and to establish the genetic relatedness. Phytochemicals were extracted according to Gomez-Romero *et al*. (2010)^63^ with slight modifications. Each dried leaf samples of *C. tora* (weight 5 g) was lyophilized in liquid nitrogen and ground to fine powder. The powder was then defatted with hexane (50 ml), and extracted using a soxhlet extractor (Quickfit, India) (30 min). Hexane was discarded through rotary evaporation. Finally, extracts were dissolved in 50 ml of methanol, incubated overnight (25 °C), filtered through 0.2 µm Millipore filter and stored (4 °C).

### FTIR

FTIR spectra were obtained from potassium bromide (KBr) pellets. The fraction (10 µl) of each extract (100 mg/ml) of *C. tora* was applied on KBr. The infrared spectra were obtained at the resolution of 1 cm^−1^ in the mid-IR range of 4,000–400 cm^−1^ using a FTIR spectrophotometer (System 2000, Perkin Elmer, Wellesley, MD, USA). All determinations were in five technical replicate, and data analyzed using statistical software OriginPro 8.0 (Fig. 3). Since, spectral reproducibility is important for creating the robust classification model; hence, variations between replicate spectra due to baseline effect, were removed by derivatization. We further obtained the FTIR spectra of standard quercetin (Supplementary Figure S1) to compare the presence of flavonoids in the tested populations of *C. tora*.

### Total flavonoid content (TFC)

TFC was determined according to Zengin *et al*. (2011)^64^. An aliquot of diluted leaf samples (1 mg/ml) as well as the standard solution of quercetin were added to 75 ml of NaNO_2_ solution, and mixed (6 min), by adding 0.15 ml AlCl_3_ (100 g/L). After 5 min, 0.5 ml of NaOH was added and the final volume adjusted to 2.5 ml with distilled water, and thoroughly mixed. Absorbance of the mixture was read at 510 nm against the blank using a UV-VIS spectrophotometer (V-550 model, Jasco, Japan). TFC concentration is expressed (mg/g dry wt) (Supplementary Table S4) based on the standard (Fig. 6). All samples were analysed in triplicate.

### Statistical analysis

FTIR-data were plotted using statistical software OriginLab (version 8.0). Differences between combined data of highland and lowland populations were analysed using a Student’s t-test analysis in SPSS software v12.0.1 (Chicago, IL, USA). Changes with P<0.05 were considered to be significant. Calibration curve of quercetin (Supplementary Figure 2) was plotted using Microsoft Office Excel (2007).

### Multivariate analysis

Polymorphic data obtained by ISSR analyses were analyzed for Bary-Curtis differentiation in the software BioDiversity Pro (version 2.0). Correlation and PCA analysis were done in PAST software (version 2.1) for clustering the transmittance data of six populations of different origins.

## Electronic supplementary material

Supplementary Information
