# Supplementary material for: Population authentication of the traditional medicinal plant Cassia tora L. based on ISSR markers and FTIR analysis
Source: Sci Rep. 2018 Jul 16;8:10714. doi: 10.1038/s41598-018-29114-1 (PMC6048050; doi:10.1038/s41598-018-29114-1)

**Title Page**

**Population authentication of the traditional medicinal plant *Cassia tora* L. based on ISSR markers and FTIR analysis**

**Author(s):** Vikas Kumar*^1^ & Bijoy Krishna Roy^1^

**Affiliation:** ^1^Centre for Advanced Study in Botany, Institute of Science, Banaras Hindu University, Varanasi -221005, India.

***Correspondence:**

Dr. Vikas Kumar

E-mail: [vk.kumarv89@gmail.com](mailto:vk.kumarv89@gmail.com)

Mobile: +91- 9670649908

**Supplementary Tables**

**Supplementary Table S1: Locations of six population of *Cassia tora* L.**

| S. N. | Population Code | Geographic Origin | Coordinates | | Altitude (m) | Annual Rainfall (mm) | Annual Temperature (°C) | Annual Solar Radiation (kWh/m^2^/day) | Voucher Specimen number |
| --- | --- | --- | --- | --- | --- | --- | --- | --- | --- |
|  |  |  | ° N | ° E |  |  |  |  |  |
| 1. | CT-1 | Dehradun, UK | 30.31 | 78.35 | 750 | 2385 | 21.4 | 5.52 | *Caesal/2014/1a* |
| 2. | CT-2 | Lucknow, UP | 26.85 | 80.94 | 123 | 724.9 | 25.2 | 4.70 | *Caesal/2014/1b* |
| 3. | CT-3 | Varanasi, UP | 25.20 | 83.00 | 80 | 934.2 | 25.8 | 5.00 | *Caesal/2014/1c* |
| 4. | CT-4 | Patna, BR | 25.60 | 85.10 | 53 | 739.9 | 25.0 | 5.22 | *Caesal/2014/1d* |
| 5. | CT-5 | Ranchi, JH | 23.35 | 85.33 | 651 | 1018 | 23.7 | 4.78 | *Caesal/2014/1e* |
| 6. | CT-6 | Puri, OR | 19.48 | 85.52 | 06 | 1467.7 | 26.9 | 4.68 | *Caesal/2014/1f* |

Data source- Indian Meterological Department

Supplementary Table S2: Pairwise genetic distance matrix computed using Jaccard’s coefficient of population of *C. tora*

|  | CT-1 | CT-2 | CT-3 | CT-4 | CT-5 | CT-6 |
| --- | --- | --- | --- | --- | --- | --- |
| CT-1 | 0 |  |  |  |  |  |
| CT-2 | 0.558 | 0 |  |  |  |  |
| CT-3 | 0.516 | 0.462 | 0 |  |  |  |
| CT-4 | 0.576 | 0.506 | 0.453 | 0 |  |  |
| CT-5 | 0.676 | 0.631 | 0.565 | 0.617 | 0 |  |
| CT-6 | 0.667 | 0.687 | 0.574 | 0.644 | 0.520 | 0 |

**Supplementary Table S3:** General band assignment of the important regions of FTIR spectrum

| Wavenumber (cm^-1^) | Functional Group | Vibration | References |
| --- | --- | --- | --- |
| 3400-3200 | -OH (Hydroxyl) | R-OH | Diem 1993; Koca et al., 2010 |
| 3000-2800 | Alkyl | C-C |
| 1800-1500 | C=C (Benzene ring)  (Acyl/Phenyl/Corbonyl group) | C=C, C=O, C-O and C-H stretching | Koca et al., 2010,  Carballo-Meilan et al., 2014 |
| 1600-1650 (1623) | Corbonyl group (Flavonoid region) | C-O and C-H stretching | Khairuddin et al., 2014 |
| 1100-900 | Aliphatic amines, Alkanes, Alkenes, | C-C, C=C, C-O and C-N stretching | Diem 1993; Carballo-Meilan et al., 2014; |

**Supplementary Table S4: Quantity of total flavonoid content of all six populations of *C. tora***

| Population Code | Absorption (nm) | Amount (mg/g) |
| --- | --- | --- |
| CT-1 | 0.190 ±0.0027* | 20.20 |
| CT-2 | 0.154 ±0.004* | 13.87 |
| CT-3 | 0.162 ±0.0045* | 15.47 |
| CT-4 | 0.161 ±0.001* | 15.20 |
| CT-5 | 0.193 ±0.0021* | 21.53 |
| CT-6 | 0.178 ±0.002* | 18.60 |

*Standard deviation

**Supplementary Table S5: Primers for RT-PCR analysis**

| **Gene** | **Acc. No. (Gene bank)** | **Forward sequence (5’-3’)** | **Reverse sequence (5’-3’)** | **Product length (bp)** |
| --- | --- | --- | --- | --- |
| ***CHS1***  ***CHS2***  ***GAPDH**** | AB015872  AB066275  CB973647 | AGCCAGTGAAGCAGGTAGCC  TCTGAGCGAGTATGGGAACA  TTCTCGTTGAGGGCTATTCCA | GTGATCCGGAAGTAGTAAT  AGGGTAGCTGCGTAGGTTGG  CCACAGACTTCATCGGTGACA | 155  294  70 |

*****Control primer

Supplementary Figures

Supplementary Figure S1: FTIR Spectrum of quercetin


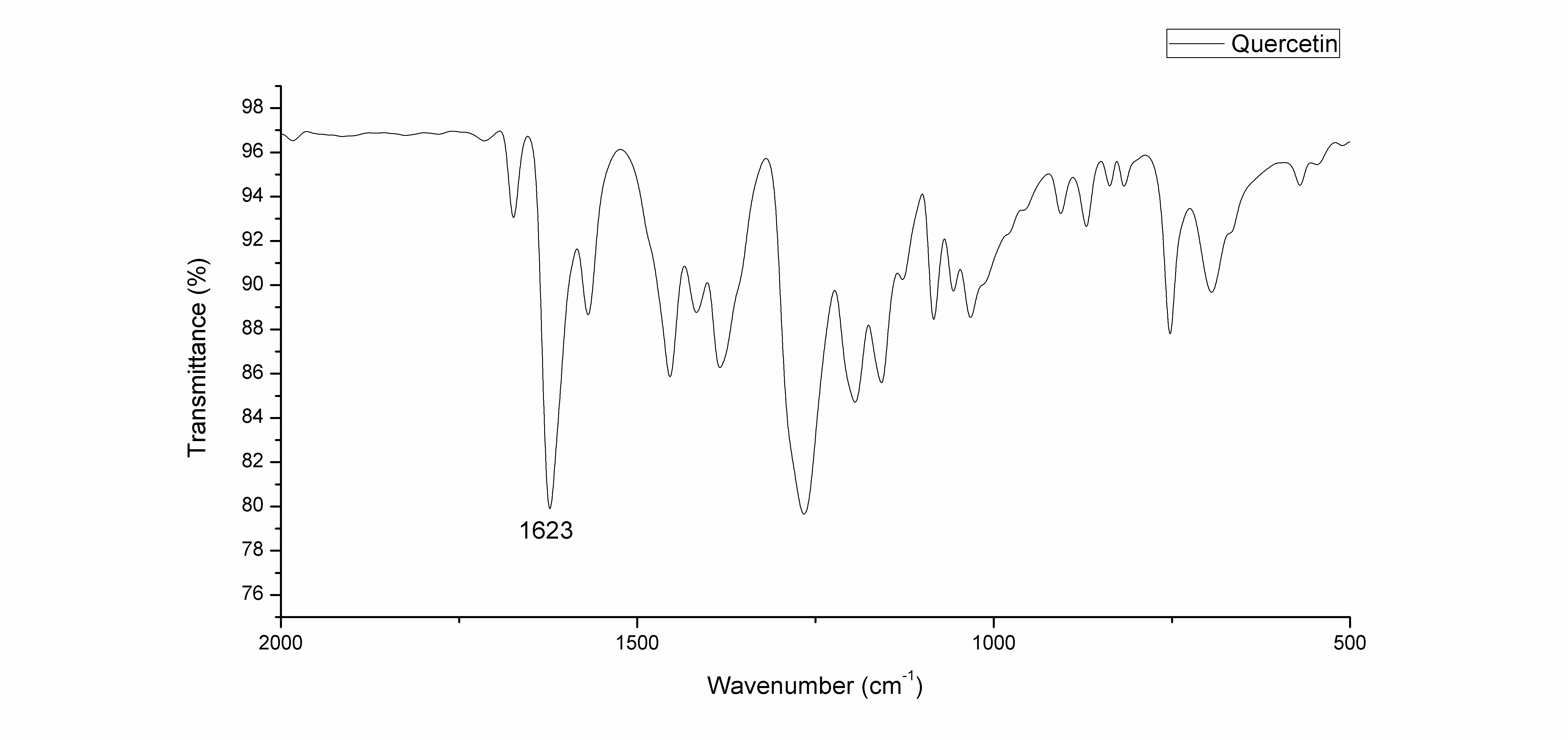


Supplementary Figure S2: Standard curve of quercetin


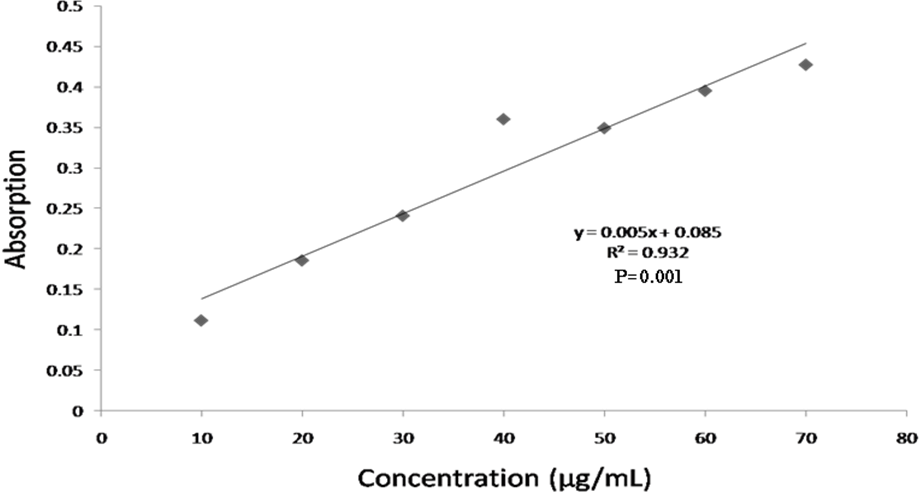

Supplement: Supplementary file 1 — Supplementary Information [file 41598_2018_29114_MOESM1_ESM.doc]
